# The Role of MMP-9 and MMP-9 Inhibition in Different Types of Thyroid Carcinoma

**DOI:** 10.3390/molecules28093705

**Published:** 2023-04-25

**Authors:** Zhenshengnan Li, Jia Wei, Bowen Chen, Yaoqi Wang, Shuai Yang, Kehui Wu, Xianying Meng

**Affiliations:** Department of Thyroid Surgery, General Surgery Center, The First Hospital of Jilin University, Changchun 130021, China; lizhensn@163.com (Z.L.);

**Keywords:** matrix metalloproteinase-9, MMP-9 inhibitor, thyroid carcinoma

## Abstract

Matrix metalloproteinase-9 (MMP-9), one of the most investigated and studied biomarkers of the MMPs family, is a zinc-dependent proteolytic metalloenzyme whose primary function is degrading the extracellular matrix (ECM). It has been proved that MMP-9 expression elevates in multiple pathological conditions, including thyroid carcinoma. MMP-9 has a detectable higher level in malignant or metastatic thyroid tumor tissues than in normal or benign tissues and acts as an additional marker to distinguish different tumor stages because of its close correlations with clinical features, such as lymph node metastasis, TNM stage, tumor size and so on. Natural and non-natural MMP-9 inhibitors suppress its expression, block the progression of diseases, and play a role in therapy consequently. MMP-9 inhibitory molecules also assist in treating thyroid tumors by suppressing the proliferation, invasion, migration, metastasis, viability, adhesion, motility, epithelial-mesenchymal transition (EMT), and other risk factors of different thyroid cancer cells. In a word, discovering and designing MMP-9 inhibitors provide great therapeutic effects and promising clinical values in various types of thyroid carcinoma.

## 1. Introduction

Matrix metalloproteinases (MMPs), named for their remarkable capacity for extracellular matrix (ECM) degradation, are a category of zin-dependent proteolytic enzymes participating in multiple physiological and pathological processes. The MMPs family consists of 28 members in vertebrates, of which at least 23 proteases are expressed in human tissues. They are generally divided into collagenases, gelatinases, stromelysins, matrilysins, membrane-type MMPs, and other MMPs, with six subtypes in total. Among this family, MMP-9 is a broadly concerned molecule of high research values and broad application prospects introduced in the review.

**Figure 1:**
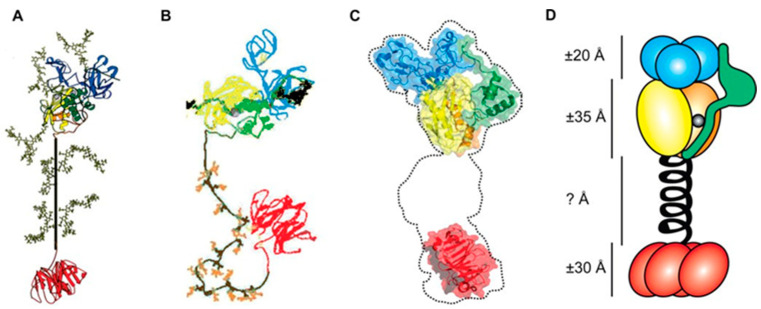
The multidomain structure of MMP-9. The propeptide is shown green, the active site is shown yellow, the three fibronectin repeats are shown blue, the metal binding site is shown orange, the catalytic zinc ion is shown grey, the OG domain is shown brown, and the PEX domain is shown red. Panels (**A**–**C**): structural models from 3 different studies. Panel (**D**): a cartoon model. (Adapted from Jennifer Vandooren et al., 2013 [1], Copyright 2013 Taylor & Francis and reproduced with permission.)

MMP-9, also known as gelatinase B, consists of five main parts as other MMPs: a signal sequence, a propeptide domain, a catalytic domain, a hinge region, and a hemopexin domain. The signal sequence guides the secretion of MMP-9 out of the cells. The propeptide domain, containing a cysteine switch motif PRCGXPD, interacts with zinc ions and prevents the combination of the water molecules to inactive MMP-9. This cysteine switch mechanism is the pivotal issue in controlling the activation of MMP-9, so MMP-3, plasmin, and other proteolytic activators activate MMP-9 through proteolysis or disruption of the propeptide domain. The catalytic domain is the vital part determining the proteolytic activity of MMP-9, formed by the zinc ion binding domain and the active site together. Here unique amino acids play a significant role in enhancing the ability and efficiency of the catalytic process. Three type-Ⅱ fibronectin repeats, similar to MMP-2, assist in the binding to elastin, collagen, gelatin, and other large substrates, becoming a modulator of collagen recognition only present in gelatinases not present in other MMPs. The specific O-glycosylated domain in the hinge region of MMP-9, also called collagen type Ⅴ-like domain on account of the similarity and homology of collagen, is unique in the MMPs family to make MMP-9 highly flexible both intradomain and interdomain. The hemopexin domain not only adds the specificity of MMP-9 in the MMPs family but also plays the following roles: interplay of substrates, combination of inhibitors, attachment of cell surface receptors, and stimulation of auto-activation [1]. The structural model and cartoon model of the multidomain structure of MMP-9 are shown in Figure 1.

Participating in growth, proliferation, apoptosis, migration, invasion, angiogenesis, and many other processes, MMP-9 is confirmed to enroll in a variety of situations clinically, such as tumors [2,3], respiratory diseases [4,5], cardiovascular diseases [6,7], cerebrovascular diseases [8,9], psychiatric disorders [10,11], ophthalmic diseases [12], autoimmune diseases [13] and so on.

## 2. MMP-9 Inhibition in Different Diseases

### 2.1. Inhibitory Mechanisms and Representative Agents of MMP-9 Inhibition

Regulating the expression of MMP-9 has been a research hot spot in recent years, and matrix metalloproteinase inhibitors (MMPIs) have been applied to multiple fields. MMP-9 is expressed and controlled in four levels: the transcription of MMP-9 genes, the translation of MMP-9 mRNAs, the proenzyme secretion from neutrophils and other cells, and the activation of the proenzyme to the active form. Based on these mechanisms, MMP-9 can be inhibited in four methods: preventing the production of MMP-9 mRNAs and proMMP-9 at the transcription and translation level, restraining the secretion of proMMP-9, blocking the activation from proMMP-9 to active MMP-9 and inhibiting the activity of active MMP-9 directly.

The first generation of MMPIs, taking batimastat and marimastat as representative agents, has a structure of hydroxamic acid, which helps to bind to zinc ions to inhibit MMP-9 activity. On account of poor solubility, low oral bioavailability, weak specificity, severe musculoskeletal syndrome, and other side effects, these hydroxamic acid derivatives mostly fail in clinical trials. The next generation of MMPIs is invented to be more targeting and efficient, including tanomastat, prinomastat, and rebimastat. However, most of them are also withdrawn from clinical trials [14].

Some MMP-9 inhibitors have poor selectivity and less efficacy, occasionally inhibiting other affinitive MMPs simultaneously because of the structural resemblance, especially the S1’ pocket of MMP-9. The molecular modeling techniques help to design selective MMP-9 inhibitors targeted to anti-cancer therapy.

Therefore, highly selective and specific MMP-9 inhibitors should be discovered and screened in natural compounds or designed by synthetic methods and applied to anti-cancer targeting therapy.

### 2.2. MMP-9 Inhibition in Different Diseases

#### 2.2.1. Natural MMP-9 Inhibitors or MMP-9 Inhibitory Molecules Applied to Different Diseases

According to a great number of studies, a variety of molecules and compounds show MMP-9 inhibitory effects, roughly divided into natural and non-natural two groups and bringing assistance to disease treatment.

Natural medicines, including plant phenolics, plant flavonoids, phytosterols, and so on, possess great potential in the treatment of tumors, ultraviolet injuries, neurodegenerative diseases, periodontal diseases, ophthalmic diseases, and other situations [15,16,17,18,19]. Compress fluids extraction, thermal processing and its alternatives, drug delivery systems, and other metabolomics techniques assist in investigating the molecular structures of natural compounds and producing the maximum medical effects [20,21]. Therefore, an intake diet of abundant nutritional natural products such as cereal grains, edible flowers and morels shows excellent benefits in health maintenance and multiple disease prevention [22].

Here we enumerate some natural MMP-9 inhibitors and molecules or compounds with MMP-9 inhibitory effects, as shown in Table 1. These natural molecules all show therapeutic effects in a wide range of diseases listed in the table based on effective drug doses.

Green tea polyphenols, the essential bioactive compounds in tea, manifests excellent potential in a variety of conditions. Additionally, among them, epigallocatechin-3-gallate (EGCG) is studied most extensively because of the most generous proportion and the most active biological activity of the total catechins. There is a strong interaction between EGCG and MMP-9, holding the galloyl group of ECG and the propeptide region of MMP-9 to be responsible, which provides them with high binding affinity in silico molecular docking analysis [23]. EGCG shows great significance in periodontal diseases, atherosclerosis, ultraviolet light oxidative damages, and, above all, multiple kinds of tumors through the inhibition of MMP-9. EGCG inhibits the activity of MMP-9 by up-regulating the cell adhesion molecules E-cadherin and β-catenin and suppressing the extracellular signal-regulated kinase (ERK) phosphorylation, restrains the proliferation, invasion, and growth of tumor cells, and becomes a promising medicine for chemoprevention or adjuvant therapy of nasopharyngeal carcinoma [24]. EGCG destabilizes the transcription of MMP-9 to down-regulate its expression in macrophage-like HL-60 myeloid leukemia cells by reducing the gene and protein level of HuR, the MMP-9 mRNA stabilizing nuclear factor. As a result, EGCG can act as an antiangiogenic agent, optimizing the current chemotherapeutic and angiosuppressive treatment of hematological malignancies [25]. EGCG reduces the up-regulated MMP-9 activity induced by ischemia, lessens neuronal damages in hippocampal CA1 and CA2 areas, shrinks the infarct volume, and protects against the ischemia-reperfusion injury, showing neuroprotective effects on transient focal cerebral ischemia [26].

**Table 1:** Natural MMP-9 inhibitors and inhibitory molecules applied to different diseases.

Reference	Molecules	Research Methods	Research Diseases	Research Subjects	Effective Dose
[18]	Morus alba Stem Extract (MSE)	ELISA RT-PCR	Periodontitis	THP-1 cells	20 µg/mL, 40 µg/mL
[24]	Epigallocatechin-3-gallate (EGCG)	Gelatin zymography	Nasopharyngeal carcinoma	TW01 cells NA cells	10 μM, 20 μM, 30 μM, 50 μM
[25]	Epigallocatechin-3-gallate (EGCG)	Gelatin zymography	Myeloid leukemia	HL-60 cells	0.3 µM, 1 µM, 3 µM, 10 µM, 30 µM
[26]	Epigallocatechin-3-gallate (EGCG)	Gelatin zymography	Transient global cerebral ischemia	A C57BL/6 mouse model of transient global cerebral ischemia	50 mg/kg
[27]	Mangiferin	Gelatin zymography RT-PCR Western blotting	Astroglioma	U87MG cells U373MG cells CRT-MG cells	30–300 μM
[28]	Mangiferin	Gelatin zymography RT-PCR Western blotting	Prostate carcinoma	LNCaP cells	400 μM
[29]	Mangiferin	Gelatin zymography RT-PCR	Glioma	U87 cells	50 μM, 100 μM
[30]	Mangiferin	ELISA Western blotting	Breast carcinoma	MDA-MB-231 cells BT-549 cells	12.5 μM, 25 μM, 50 μM
[30]	Mangiferin	Western blotting	Breast carcinoma	An SCID mouse MDA-MB-231 xenograft model	100 mg/kg
[31]	β-sitosterol (SITO)	Western blotting	Colon carcinoma	CT26/luc cells	16 µM
[31]	Liposomal β-sitosterol (LS)	Western blotting	Colon carcinoma	CT26/luc cells	16 µM
[32]	Resveratrol (RES)	Gelatin zymography	Vascular leakage	THP-1 cells	30 μM
[32]	Resveratrol-Linoleate (RES-LA)	Gelatin zymography	Vascular leakage	THP-1 cells	10 μM, 20 μM, 30 μM, 40 μM
[33]	Salvianolic acid A (SAA)	Western blotting	Ischemia reperfusion	An SD rat model of cerebral ischemia reperfusion	5 mg/kg, 10 mg/kg, 20 mg/kg
[34]	Theaflavin	RT-PCR	Periodontitis	A Wistar rat model of ligatured periodontitis	10 mg/mL, 100 mg/mL
[35]	Curcumin	RT-PCR Western blotting	Atherosclerosis (AS)	THP-1 cells	6.25 µM, 25 µM, 50 µM
[36]	β-elemene	Immunohistochemistry RT-PCR Western blotting	Melanoma	A C57BL/6J mouse model with subretinal injection of B16F10 cells	2 μL every 2 days
[37]	Angelica gigas (AG)	RT-PCR	Periodontitis	An SD rat model of ligature-induced periodontitis	1 mg/mL, 100 mg/mL
[37]	Angelica gigas (AG)	ELISA	Periodontitis	HDF cells	1 µg/mL, 10 µg/mL, 100 µg/mL

ELISA: enzyme linked immunosorbent assay; RT-PCR: reverse transcription-polymerase chain reaction.

Mangiferin is a natural polyphenol compound widely derived from higher plants such as Mangifera indica and broadly applied to many diseases. Mangiferin exhibits numerous beneficial pharmacological properties, including antioxidant, anti-inflammatory, anti-diabetic, anti-cancer, and analgesic effects, and some of these functions come to work by the inhibition of MMP-9. Mangiferin suppresses MMP-9 activity in human astroglioma cells at the promoter, mRNA, and protein levels through the inhibition of nuclear factor-κB (NF-κB) and AP-1 binding to the MMP-9 promoter and the down-regulated phosphorylation of Akt and MAP kinases, two upstream signaling molecules of MMP-9. Thus, mangiferin is considered a valuable medical natural product for the therapy of malignant gliomas [27]. Mangiferin inhibits MMP-9 expression in prostate cancer cells by suppressing NF-κB activity [28], inhibits MMP-9 expression in glioma cells by promoting microRNA-15b (miR-15b) level [29], and inhibits MMP-9 expression in breast cancer cells by weakening the activation of β-catenin pathway and reversing the process of epithelial-mesenchymal transition (EMT) [30]. These inhibitory results verify the great anti-tumor potential of mangiferin as an effective chemopreventive agent used in different kinds of tumors.

Various natural molecules possess MMP-9 inhibitory effects. However, high melting point, high molecular weight, poor water solubility, poor gastrointestinal permeability, poor stability, and other shortages limit their bioavailability. Based on the dilemma, liposomes, nanoparticles, and other drug delivery systems have been used to modulate the pharmacokinetics and enhance the targeting ability of the compounds.

β-sitosterol (β-SITO), a member of the phytosterol family, owns various biological properties such as anti-inflammatory, anti-cancer, anxiolytic, cholesterol-lowering, and has been applied to anxiety, diabetes, rheumatoid arthritis (RA), a variety of cancers and many other diseases. However, its poor water solubility and bioavailability make the treatment less effective. Liposomes, hollow spheres consisting of phospholipid bilayers, are usually served as efficient drug delivery vehicles because of the advantage of increasing drug stability, prolonging the circulation time, improving specific uptake, and enhancing water solubility. Liposomal encapsulated β-SITO (LS), synthesized by the thin-film hydration method using egg phosphatidylcholine (EPC), shows better invasion inhibition, lower cytotoxicity, and fewer metastases to other tissues compared with SITO when treating colon carcinoma. The inhibition of MMP-9 and the elicitation of anti-tumor immune response generated by LS further prove it to be a more promising anti-tumor drug [31].

Resveratrol (RES), a natural stilbenoid similar to β-SITO, shows great anti-oxidant and anti-cancer effects as well as poor pharmacokinetics, potency, and bioavailability. Novel RES-lipid conjugates, such as resveratrol-linoleate (RES-LA), are lipophilic derivatives of RES synthesized by enzymatic and chemical synthesis. RES-LA represents better lipophilicity and bioavailability, more substantial cell membrane penetration, and greater inhibition of MMP-9 by inactivating ERK1/2 and JNK1/2 MAP kinase signaling pathways compared with RES in the inflammatory treatment. As a result, RES-LA is used to preserve the connecting integrity between endothelial cells, reduce pathological endothelial permeability and mitigate vascular endothelial dysfunction under inflammation [32].

These circumstances suggest that natural products indeed possess MMP-9 inhibitory effects in plenty of disorders. Novel natural molecule conjugates loading on drug delivery systems should be designed and synthesized to improve the bioavailability and enhance the inhibition of MMP-9 with a broad treating application prospect.

#### 2.2.2. Non-Natural MMP-9 Inhibitors or MMP-9 Inhibitory Molecules Applied to Different Diseases

Another group of MMP-9 inhibitors is non-natural synthetic molecules, as shown in Table 2.

SB-3CT, a potent and selective synthetic inhibitor targeting MMP-2 and MMP-9, is commonly used in brain damage caused by various reasons. Through antagonizing the increasing level of MMP-9, SB-3CT provides behavioral protection, preserves hippocampal neurons, prevents cell apoptosis, attenuates brain infarct volumes, improves neurobehavioral outcomes, and ameliorates neurobehavioral prognosis of brain injuries caused by trauma, embolism and hypoxia [38]. In addition to cerebrovascular diseases, SB-3CT shows anti-tumor ability in melanoma and lung cancer by inhibition of MMP-9 through the reduction of programmed death ligand 1 [66] and anti-inflammation ability in inflammatory corneal neovascularization by suppression of MMP-9 and VEGF-C together to reduce corneal lymphangiogenesis and macrophage infiltration [40].

According to pharmacokinetics studies, SB-3CT is poorly water soluble, and it primarily metabolizes to p-hydroxy SB-3CT on account of the oxidation of the terminal phenyl, which shows a more powerful and efficient ability to inhibit MMP-9. To enhance the inhibitory effects, an innovative prodrug strategy emerges for better water solubility and therapeutic outcomes. That is synthesizing the O-phosphate prodrug of p-hydroxy SB-3CT. This prodrug has few effects of its own but hydrolyzes to p-hydroxy SB-3CT in human blood and crosses the blood-brain barrier (BBB) easily, making therapeutic significance in cerebral diseases [67].

A portion of synthetic MMP-9 inhibitors is designed to target two or more enzymes simultaneously because of their similar structures and functions. AZD1236, a selective oral inhibitor of MMP-9 and MMP-12, has been applied to the treatment of spinal cord injury through suppression of edema, pain sensation, blood-spinal cord barrier (BSCB) breakdown, and improvement of electrophysiological, sensory, and motor functions [41]. It can also be used in the adjuvant therapy of moderate to severe chronic obstructive pulmonary disease (COPD) safely and tolerably [42,68]. AZ11557272, a dual MMP-9 and MMP-12 inhibitor, abolishes smoke-induced lavage inflammatory cells and serum tumor necrosis factor α (TNFα), ameliorates morphological emphysema targets of COPD, and participates in the therapeutic process [43]. AQU-118, an oral inhibitor of MMP-2 and MMP-9, reduces mechanical allodynia and caspase-3 expression in the dorsal root ganglion (DRG) of rats to treat spinal nerve ligation-induced neuropathic pain. More research should be performed to determine whether AQU-118 is efficient and applicable to treating neuropathic pain in humans [69].

The majority of MMP-9 inhibitors are designed to bind to the highly conserved enzyme catalytic active site of MMP-9. However, this kind of inhibitory mechanism fails in clinical trials mainly because of low specificity and high toxicity, for the inhibitors not only bind to the zinc ion of MMP-9 but also combine with other heavy metals in different proteins.

The N-terminal domain of TIMP-2 (N-TIMP2), remaining strong inhibitory effects of various MMPs, is chosen as a scaffold to design novel high-affinity and high-specificity MMP inhibitors. Jason Shirian constructs a small combination library of abundant N-TIMP2 mutants and selects N-TIMP2 variants targeting MMP-9 and MMP-14, using computational design and yeast surface display (YSD) technology. The specific inhibitor, converted from a broad inhibitor, exhibits a thousand-fold binding capacity over other MMPs and is a successful example of diagnostic and therapeutic tools targeting MMP-9 to inhibit the development of diseases [70].

Differing from the mechanisms of conventional MMP-9 inhibitors, novel inhibitors describe innovative and characteristic inhibitory mechanisms, showing a promising application prospect clinically. (I-3, II-3)-biacacetin is a natural product-like biflavone synthesized by one-pot protocol, an oxidative dimerization synthesis of poly-substituted diaryl-1,3-diketones and cerium ammonium nitrate. The molecule shows no hydrogen bonding with MMP-9 but hydrophobic interactions with the amino acid residues and binds outside the S10 active site instead of interacting with the zinc ion in the catalytic site, in silico docking analysis [44].

JNJ0966 is a highly selective and safe MMP-9 inhibitor. The compound interacts with a structural pocket in proximity to the MMP-9 zymogen cleavage site near Arg-106, distinct from the catalytic domain, inhibits the activation of MMP-9 zymogen and prevents the conversion to the catalytically active enzyme. JNJ0966 has obtained prominent curative effects in the mouse model of autoimmune encephalomyelitis, which represents a significant advance in the field of MMP-9 inhibition use in clinical treatment [71].

#### 2.2.3. MMP-9 Inhibitors or MMP-9 Inhibitory Molecules Involved in Clinical Trials

MMP-9 inhibitors have been used in clinical trials a great many times, experiencing many failures. Currently, several phase 1 and phase 2 clinical trials have obtained a few results. Curcumin is involved in a randomized controlled clinical trial designed for investing the effects on MMP-9 in patients with coronary artery diseases. The results show that curcumin can prevent cardiovascular diseases by declining the expression and activity of MMP-9 [72]. GS-5745, an anti-MMP-9 monoclonal antibody, is involved in a phase 1 randomized clinical trial to evaluate the safety and efficacy of treating ulcerative colitis [73]. COL-3, an MMP inhibitor, is involved in a phase 1 clinical trial to determine its doses and toxicities in patients with refractory metastatic tumors [74]. Andecaliximab, an inhibitor of MMP-9, is involved in a phase 2 randomized open-label clinical trial to estimate the therapeutic effects on advanced gastric carcinoma, comparing using nivolumab alone with using nivolumab plus andecaliximab together. The combination treatment of these two drugs has a favorable safety with no more remarkable effects than the single treatment [75].

## 3. MMP-9 Expression in Thyroid Carcinoma

### 3.1. Introduction of Thyroid Carcinoma Diagnosis

Thyroid carcinoma (TC), the most common endocrine cancer, has a rising incidence and mortality increased by 60–200% during nearly 30 years from 1990 to 2017, with an apparent higher incidence in females than in males, revealing a distinct epidemiological pattern [76].

Thyroid tumors are classified into several categories: thyroid nodules, mostly small, non-palpable and benign lesions; differentiated thyroid cancer (DTC), originating from thyroid epithelial cells and accounting for more than 95% of malignant cases; anaplastic thyroid cancer (ATC), a rare but dangerous form with a high possibility of metastasis, accounting for 1%; medullary thyroid cancer (MTC), originating from the parafollicular neuroendocrine cells of thyroid and accounting for 1–2% [77]. Papillary thyroid cancer (PTC) is the leading histological subtype of overall thyroid tumors, followed by follicular thyroid cancer (FTC). The incidence of PTC increased visibly and systematically with large variability by sex in the 25 observed countries during the period from 1998 to 2012 [78].

Fine-needle aspiration (FNA) is a confirmatory detection test for the diagnosis of thyroid cancer, with high sensibility and high specificity, widely accepted and used around the world. However, this technique highly depends on the standard meticulous operation and considerable experience of operators [79]. In addition, it is an invasive detection with possibilities of misdiagnosis, risks of hemorrhage and metastasis after the procedure, and feelings of pain and anxiety [80].

In order to develop a new safe, and accurate diagnosis approach, tumor biomarkers receive lots of attention. Calcitonin levels in medullary thyroid cancer, BRAFV600E mutations in papillary thyroid cancer, RAS mutations in follicular thyroid carcinoma, and levels of thyroglobulin (TG) and thyroid stimulating hormone (TSH) are all significant biomarkers.

This review describes MMP-9 as a tumor marker for thyroid cancer, for this protein is involved in multiple aspects of the progression of the tumor.

### 3.2. MMP-9 Expression Levels in Thyroid Carcinoma

According to a variety of studies, MMP-9 expresses higher in thyroid tumor tissues than in non-tumor tissues, in malignant tumors than in benign tumors, in metastatic tumors than in non-metastatic tumors, and before operation than after operation in general, mainly detected by immunohistochemistry (IHC), gelatin zymography, western blotting, reverse transcription-polymerase chain reaction (RT-PCR) and enzyme linked immunosorbent assay (ELISA), as shown in Table 3.

Therefore MMP-9, showing high malignant potential in thyroid carcinoma, can be considered as a kind of biomarker assisting in diagnosing the pathological type of tumors, assessing the metastatic status, and evaluating the therapeutic effects of the operation.

Various types of research show close correlations between MMP-9 levels and clinicopathological characteristics, such as lymph node metastasis, extrathyroidal invasion, degree of tumor infiltration, TNM stage, tumor size, distant metastasis, age, and lymphovascular invasion, as shown in Table 4.

Jelena Roncevic observes that high levels of MMP-9 in PTC patients with unfavorable clinicopathological features, including the presence of lymph node metastasis, extrathyroidal invasion, large tumor size, and advanced TNM stage, which confirms the important role of MMP-9 in the progression of tumor and the prognosis of patients [81]. Xingkai Liu discovers that PTC patients with central and lateral lymph node metastasis have an increasing level of MMP-9 and an advanced TNM stage, making MMP-9 assessment a supplementary diagnosing and predicting tool clinically [82]. Another research proves that only active patterns of MMP-9, rather than the total levels of MMP-9, have significant associations with lymph node metastasis, extrathyroidal invasion, and degree of tumor infiltration. The ratio of MMP-9 activation also plays an important role, providing new ideas for the detection of active MMP-9 instead of total MMP-9 in a further study [83].

Some researchers have analyzed the clinical significance of MMP-9 levels to predict the prognosis of thyroid carcinoma in longitudinal studies. As for the prognosis of diseases, two investigation objects are taken into analysis generally, recurrence and survival time without structurally persistent/recurrent disease (SPRD). After a period of no evidence of the disease, unexpected structural diagnostic evidence is named recurrence. The period from premier treatment to the occurrence of structurally persistent disease, recurrent disease, or last follow-up is investigated as survival time without SPRD. Dahai Xu finds a significantly higher MMP-9 score in patients with SPRD than without SPRD, representing MMP-9 as a potential and supplementary tool for prognosis prediction [84]. Daniel Buergy detects that increasing levels of MMP-9 are consistent with the metastasis of the tumor and the tumor recurrence locally, which indicates its predictive relevance to tumor progression and prognosis [85].

MMP-9 activity can also distinguish sporadic PTC and radiation-associated PTC using immunochemistry clinically. Patients diagnosed with PTC on account of exposure to the external and internal radiation of the Chernobyl nuclear accident have an abundant expression of MMP-9 in tumor tissues, demonstrating the high aggression of the tumor [86]. The increased incidence of thyroid cancer after the accident results from iodine deficiency in the contaminated territories, contributing to more frequent thyroid goiter and thyroid nodules.

Besides being relevant to clinicopathological factors alone, MMP-9 also shows significant relationships with other molecules. There is a statistically significant correlation between the expression of MMP-9 and VEGF-C, the simultaneous levels of which have significant effects on lymph node metastasis, TNM stage, and degree of tumor infiltration. This condition explains that MMP-9 contributes only a few parts of the tumor progression and generally works with other molecules together to regulate the tumors [87].

Although a majority of research has supported that MMP-9 expression levels are relevant to high metastatic potential and poor prognosis closely, there are still several studies that find no statistical significance between MMP-9 and the metastasis of tumors [88,89,90].

**Table 3:** MMP-9 expression levels in thyroid carcinoma.

Reference	Type of Thyroid Carcinoma	Type of Samples	Research Objects	Research Methods	MMP-9 Expression Data	MMP-9 Expression Levels
[91]	PTC	Tissue	86 patients with PTC	Immunohistochemistry Gelatin zymography Western blotting	IHC positive staining ratio: PTC tumor tissues: 71/86 (82.56%) PTC non-tumor tissues: 53/86 (61.63%)	MMP-9 had a statistically significant higher level in tumor tissues than non-tumor tissues in PTC (*p* < 0.05).
[92]	PTC	Tissue	25 patients with non-metastatic small PTC 19 patients with metastatic small PTC	Immunohistochemistry	IHC scores: Non-metastatic PTC tumor tissues: 1.44 Non-metastatic PTC non-tumor tissues: 0.80 Metastatic PTC tumor tissues: 2.00 Metastatic PTC non-tumor tissues: 0.82	MMP-9 had a statistically significant higher level in tumor tissues than non-tumor tissues in both non-metastatic PTC (*p <* 0.01) and metastatic PTC (*p* < 0.001).
[93]	PTC	Tissue	83 patients with PTC	Immunohistochemistry RT-PCR	IHC positive staining ratio: PTC tumor tissues: 48/83 (57.83%) PTC non-tumor tissues: 2/83 (2.41%)	MMP-9 had a statistically significant higher level in tumor tissues than non-tumor tissues in PTC (*p <* 0.001).
[94]	PTC	Tissue	50 patients with PTC	Immunohistochemistry RT-PCR	/	MMP-9 had a statistically significant higher level in tumor tissues than non-tumor tissues in PTC (*p* = 0.01).
[95]	PTC	Tissue	60 patients with PTC 30 patients with MNG	ELISA RT-PCR	mRNA level: PTC tumor tissues: 8.05 ± 16.48 PTC non-tumor tissues: 3.06 ± 3.63 Protein level: PTC tumor tissues: 429.60 ± 288.54 PTC non-tumor tissues: 223.15 ± 137.68	MMP-9 had a statistically significant higher level in tumor tissues than non-tumor tissues in PTC (*p <* 0.05).
[82]	PTC	Tissue	112 patients with PTC 42 patients with BTN	Immunohistochemistry	IHC scores: PTC tumor tissues: median 4.0, IQR 2.0–8.0 BTN tumor tissues: median 1.0, IQR 0.0–1.0	MMP-9 had a statistically significant higher level in PTC tissues than in BTN tissues (*p <* 0.001).
[84]	PTC	Serum	182 patients with PTC 86 patients with BTN 62 HCs	ELISA	Protein level: PTC serums: median 79.45, IQR 64.06–113.15 BTN serums: median 47.35, IQR 38.05–68.14 HC serums: median 47.71, IQR 36.70–59.52	MMP-9 had a statistically significant higher level in PTC serums than in BTN serums (*p <* 0.001) and HC serums (*p <* 0.001).
[85]	PTC MTC FTC ATC	Tissue	47 patients with TC 22 patients with FA	ELISA	/	MMP-9 had a statistically significant higher level in TC tissues than in FA tissues (*p* = 0.001).
[95]	PTC	Tissue	60 patients with PTC 30 patients with MNG	ELISA RT-PCR	mRNA level: PTC tumor tissues: 6.77 ± 7.16 MNG tumor tissues: 2.49 ± 3.70 Protein level: PTC tumor tissues: 429.60 ± 288.54 MNG tumor tissues: 218.14 ± 113.74	MMP-9 had a statistically significant higher level in PTC tissues than in MNG tissues (*p <* 0.05).
[96]	FTC	Tissue	6 patients with WIFC 15 patients with MIFC 19 patients with FA 10 patients with AG	Immunohistochemistry	IHC positive staining ratio: MIFC tumor tissues: 13/15 (86.67%) FA tumor tissues: 11/19 (57.89%) AG tumor tissues: 3/10 (30.00%)	MMP-9 had a statistically significant higher level in MIFC tissues than in FA tissues (*p <* 0.05) and AG tissues (*p <* 0.005).
[97]	PTC	Tissue	66 patients with PTC 40 patients with BTN	Immunohistochemistry	IHC positive staining ratio: PTC tumor tissues: 61/66 (92.42%) BTN tumor tissues: 8/40 (20.00%)	MMP-9 had a statistically significant higher level in PTC tissues than in BTN tissues (*p <* 0.001).
[98]	DTC	Serum	57 patients with DTC 49 patients with BTN 20 HCs	ELISA RT-PCR	Protein level: Mean ± SD DTC serums: 134.70 ± 32.52 BTN serums: 47.60 ± 20.10 HC serums: 40.52 ± 10.20	MMP-9 had a statistically significant higher level in DTC serums than in BTN serums (*p <* 0.05) and HC serums (*p <* 0.05).
[99]	PTC	Serum	41 patients with PTC 56 patients with BTN	ELISA	Protein level: PTC serums: 299.98 ± 70.48 BTN serums: 126.62 ± 19.26	MMP-9 had a statistically significant higher level in PTC serums than in BTN serums (*p <* 0.01).
[100]	PTC	Plasma	30 patients with PTC 30 patients with BTN 23 HCs	ELISA	Protein level: Mean ± SD PTC plasma: 72.3 ± 23.3 BTN plasma: 23.0 ± 2.2 HC plasma: 22.1 ± 3.0	MMP-9 had a statistically significant higher level in PTC plasma than in BTN plasma (*p <* 0.05) and HC plasma (*p <* 0.05).
[101]	PTC FTC ATC	Cell	IHH-4 cells FTC-133 cells 8505C cells HT-ori3 cells	Western blot RT-PCR	Protein level (the relative gray value): HT-ori3 cells: 1.01 ± 0.43 IHH-4 cells: 6.59 ± 1.24 FTC-133 cells: 5.10 ± 0.91 8505C cells: 5.42 ± 0.86 mRNA level: HT-ori3 cells: 1.01 ± 0.09 IHH-4 cells: 4.56 ± 0.61 FTC-133 cells: 3.41 ± 0.42 8505C cells: 2.79 ± 0.26	MMP-9 had a statistically significant higher level in IHH-4 cells, FTC-133 cells, and 8505C cells than HT-ori3 cells (*p <* 0.05).
[98]	DTC	Serum	57 patients with DTC 49 patients with BTN 20 HCs	ELISA RT-PCR	Protein level: DTC preoperative serums: 134.70 ± 32.52 DTC postoperative serums (1 month after surgery): 51.46 ± 18.34	MMP-9 had a statistically significant higher level in DTC serums before operation than after operation (*p <* 0.05).
[99]	PTC	Serum	41 patients with PTC 56 patients with BTN	ELISA	Protein level: PTC preoperative serums: 299.98 ± 70.48 PTC postoperative serums (3 months after surgery): 201.65 ± 65.31 PTC postoperative serums (6 months after surgery): 184.64 ± 64.82 PTC postoperative serums (12 months after surgery): 169.07 ± 64.16	MMP-9 had a statistically significant higher level in PTC serums before operation than after operation (*p <* 0.05).
[102]	PTC	Tissue	27 patients with metastatic PTC 31 patients with non-metastatic PTC	Immunohistochemistry	IHC positive and strongly positive staining ratio: Metastatic PTC tumor tissues: 19/27 (70.37%) Non-metastatic PTC tumor tissues: 11/31 (35.48%)	MMP-9 had a statistically significant higher level in PTC tumor tissues with metastasis than without metastasis (*p <* 0.05).
[103]	PTC	Tissue	71 patients with cervical lymph node metastatic PTC 85 patients with non-metastatic PTC	Immunohistochemistry	IHC intense positive staining ratio: Metastatic PTC tumor tissues: 17/21 (80.95%)Non-metastatic PTC tumor tissues: 4/21 (19.05%)	MMP-9 had a statistically significant higher level in PTC tumor tissues with metastasis than without metastasis (*p* = 0.000).
[104]	PTC	Tissue	17 patients with CPTC 25 patients with FPTC	Immunohistochemistry	IHC positive staining ratio: FPTC tumor tissues: 20/25 (80.00%) CPTC tumor tissues: 17/17 (100.00%)	MMP-9 had a statistically significant higher level in CPTC tissues than in FPTC tissues (*p <* 0.004).

PTC: papillary thyroid cancer; FTC: follicular thyroid cancer; MTC: medullary thyroid cancer; ATC: anaplastic thyroid cancer; MNG: multinodular goiter; WIFC: widely invasive FTC; MIFC: minimally invasive FTC; FA: follicular adenoma; AG: adenomatous goiter; BTN: benign thyroid nodule; HC: healthy control; CPTC: classical PTC; FPTC: follicular PTC.

**Table 4:** Correlations between MMP-9 levels and clinicopathological characteristics.

Reference	Type of Thyroid Carcinoma	Type of Samples	Lymph Node Metastasis (LNM)	Extrathyroidal Invasion (EI)	Degree of Tumor Infiltration (DTI)	TNM Stage	Tumor Size	Distant Metastasis	Age	Lymphovascular Invasion
[81]	PTC	Tissue	*p* = 0.028	*p* = 0.001		*p* = 0.005	*p* = 0.031			
[82]	PTC	Tissue	Central LNM *p* = 0.002 Lateral LNM *p <* 0.001			*p* = 0.004				
[83]	PTC	Tissue	*p* = 0.004	*p <* 0.001	*p <* 0.001				*p* = 0.034	
[91]	PTC	Tissue	*p* = 0.014							
[93]	PTC	Tissue	*p <* 0.001 (protein) *p* = 0.038 (mRNA)				*p* = 0.008			
[94]	PTC	Tissue		*p* = 0.019	*p* = 0.019					
[95]	PTC	Tissue				*p* = 0.011 (mRNA) *p* = 0.001 (protein)			*p* = 0.015 (mRNA) *p* = 0.001 (protein)	*p* = 0.003 (mRNA) *p* = 0.036 (protein)
[97]	PTC	Tissue				*p* = 0.006	*p <* 0.001		*p* = 0.003	
[105]	FTC	Tissue					*p* = 0.001	*p* = 0.016		
[84]	PTC	Serum	Lateral LNM *p <* 0.001	*p* = 0.022		*p <* 0.001	*p* = 0.029	*p* = 0.003		
[98]	DTC	Serum	*p <* 0.05		*p <* 0.05	*p <* 0.05	*p <* 0.05			
[100]	PTC	Plasma	*p <* 0.001	*p* = 0.037		*p* = 0.003	*p* = 0.002	*p* = 0.034		

These results may be due to the following several reasons: 1. The detection methods of MMP-9, such as immunohistochemistry, are mostly semi-quantitative, requiring the subjective judgments of researchers, which may have different influences on the final results. 2. The research objects are different in different studies. Some studies focus on the differences between metastatic and non-metastatic tumors rather than malignant tumors and benign disease, which shows inconsistency in the final conclusions.

Coincidentally, Ruxandra Dobrescu draws a conclusion that serum MMP-9 levels not only show no differences between benign and malignant patients but also differ insignificantly in ages, histological subtypes, TNM stages, multifocal or unifocal tumors, small or large tumor sizes, lymph node metastasis, and extrathyroidal extension. As for these contradictory results compared to previous studies, researchers demonstrate four reasons: 1. The most representative and most substantial evidence comes from MMP-9 detection in tumor tissues, and the serum levels only partly reflect the tumor situation. 2. Increasing levels of MMP-9 are involved in various processes containing inflammation, autoimmune disorders, and degenerative diseases, which should also be taken into account. 3. ELISA technology measures the total of proMMP-9, active MMP-9, degradation products, and other complexes instead of a single molecule. 4. The study uses serums as samples, which may provide different results from plasma samples because of coagulants and anticoagulants [106].

MMP-9 participates in a series of signaling pathways in thyroid carcinoma, as shown in Table 5. Possessing a steadily increasing incidence, thyroid cancer has relatively low mortality on the contrary compared with other malignant tumors. Hence the most significant and challenging focus is not operating a perfect thyroidectomy operation on all patients but treating them by grading diagnosis and grading therapy in order to avoid overtreatment of patients with low risks and identify advanced patients with high stakes, metastatic potential, and poor prognosis [77].

The detection of MMP-9 levels plays a vital role in this matter. In most cases, high MMP-9 levels in tumor tissues or serums relate to an advanced stage and high risks, and patients in these circumstances need more aggressive and rapid treatment approaches. In other words, adding MMP-9 detection to preoperative examinations may assist in grading the patients who require different patterns of therapy, such as thyroidectomy surgery or radioiodine therapy. Additionally, a quick and accurate MMP-9 detection during the operation period time may help surgeons judge the surgery forms and scopes, such as lymph node dissection area and the extent of surgical thyroid resection.

## 4. MMP-9 Inhibition in Thyroid Carcinoma

### 4.1. Introduction of Thyroid Carcinoma Therapy

The treatment of thyroid cancer is a multidisciplinary cooperative process. Thyroidectomy in the open field or under the endoscope is the most common method to treat differentiated thyroid carcinoma, sometimes accompanied by the problem of overtreatment. Radioactive iodine therapy is a preferred adjuvant therapy for remnant ablation and recurrent tumor administered after surgery, with an increased risk of secondary malignancies development. TSH suppression therapy is chiefly used in high-risk patients with incomplete resection, tumor invasion, or distant metastasis, which may lead to side effects such as osteoporosis. Chemotherapy after surgery is a treatment alternative, mostly for poorly differentiated anaplastic cancer. Doxorubicin, sorafenib, axitinib, and other chemotherapeutic agents have shown some promise of the tumor holding a very poor prognosis [120,121].

Apart from the above treatment methods, MMP inhibitors, especially inhibitors for MMP-9, which have been confirmed to link with thyroid cancer, can act as targeting drugs with promising prospects for future development. These inhibitory molecules or compounds exhibit specific targeting ability in the signaling pathways of tumors and have achieved good curative effects in vivo and in vitro, which can be an effective supplementary therapy for thyroid cancer.

### 4.2. MMP-9 Inhibition in Thyroid Carcinoma

#### 4.2.1. Natural MMP-9 Inhibitors or MMP-9 Inhibitory Molecules Applied to Thyroid Carcinoma

**Figure 2:**
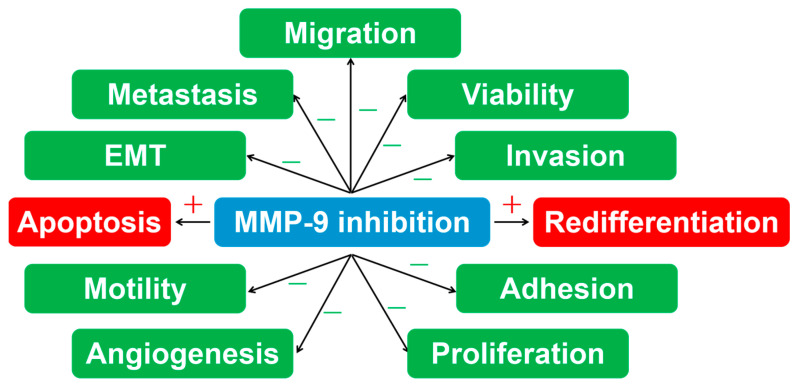
The mechanisms of MMP-9 inhibition in thyroid tumor cells.

Similar to MMP-9 inhibitors applied to different diseases and situations, MMP-9 inhibitors and inhibitory molecules also work in thyroid carcinoma and can be divided into two groups, natural and non-natural, as shown in Table 6 totally. The mechanisms of MMP-9 inhibition in different aspects are shown in Figure 2.

Curcumin, a constituent of the traditional medicine known as turmeric, shows potential therapeutic effects on various diseases such as cardiovascular diseases, aging-related diseases, and a wide range of cancers, including colorectal cancer, breast cancer, and thyroid cancer certainly. Curcumin inhibits the metastasis of K1 papillary thyroid cancer cells via down-regulating the expression of MMP-9 and up-regulating the expression of E-cadherin, which consequently suppresses the epithelial-mesenchymal transition (EMT) and reduces the viability and migration of tumor cells [122]. Moreover, hypoxia, a common condition essential for tumor metastasis, stimulates the expression of transcription factor hypoxia-inducible factor 1 (HIF-1) in tumor cells and activates many genes of proteases, including MMP-9. Curcumin decreases reactive oxygen species (ROS) induced by hypoxia, suppresses the expression of HIF-1 in K1 cancer cells, enhances E-cadherin expression, and inhibits MMP-9 activity. As a result, curcumin alleviates the migration of K1 cells under hypoxic conditions, showing anti-cancer and anti-metastasis functions in PTC [123]. MicroRNAs (miRNAs or miRs) participate in a variety of tumors, acting as either oncogenes or tumor suppressors. Curcumin up-regulates miR-301a-3p, which directly targets STAT3 (signal transducer and activator of transcription 3) and suppresses its expression. MiR-301a-3p, a tumor suppressor, targets STAT3 directly and suppresses the protein expression. By regulating the miR-301a-3p/STAT3 axis, curcumin increases the expression of miR-301a-3p, decreases the expression of MMP-9 and EMT markers, and inhibits the viability, migration, and invasion of TPC-1 papillary thyroid cancer cells [124]. These consequences all prove that curcumin can be regarded as a high-efficiency and high-safety natural MMP-9 inhibitor, exhibiting great significance in the treatment of papillary thyroid cancer in many aspects.

Quercetin is one of the most abundant polyphenolic flavonoids present in grapefruits, onions, berries, and other fruits and vegetables. Applied to the therapy of plenty of diseases, quercetin shows vast therapeutic potential, such as anti-viral, anti-cancer, anti-oxidant, anti-diabetic, and so on. NIS (Sodium iodide symporter) mediates the accumulation of iodide in thyrocytes and promotes ablative therapy with radioiodine. A reduction of NIS in some PTC patients leads to low effective radioiodine therapy. Quercetin increases the expression and function of NIS, inhibits MMP-9 activity, and enhances E-cadherin activity so as to achieve strong therapeutic effects in the process of the suppression of cell migration and EMT [133]. As a consequence, thyroid cancer patients who need radioiodine treatments are suggested to take quercetin as a kind of adjuvant medicine to improve the curative effects.

Panax ginseng is a well-known Chinese traditional herb usually used as a kind of tonic, and ginsenoside is the bioactive constituent extracted from the root or stem of it. Ginsenoside has many beneficial pharmacological properties, especially anti-metastatic and anti-inflammatory effects intimately related to the inhibition of MMPs through modulating the signaling pathways [141]. For example, ginsenoside Rg1 suppresses the invasion and migration of breast cancer cells mainly via the down-regulation of MMP-9, which is mediated by NF-κB [142]. When used in thyroid cancer, ginsenoside Rg3 shows metastasis suppression effects mainly through the mechanism of alternating actin skeleton of tumor cells and destroying the function. Ginsenoside Rg3 also decreases the levels of MMP-2, MMP-9, Rac-1, and Cdc42, blocking lymph node metastasis in PTC and angiogenesis in ATC [128]. As a consequence, ginsenoside compounds can be considered as MMP-9 inhibitors applied to treat different kinds of disorders, including thyroid cancer.

#### 4.2.2. Non-Natural MMP-9 Inhibitors or MMP-9 Inhibitory Molecules Applied to Thyroid Carcinoma

Apart from natural MMP-9 inhibitors, there are some non-natural molecules possessing MMP-9 inhibitory abilities in thyroid cancer cells, although they are not mainly used in the treatment of thyroid carcinoma.

Differentiation therapy is a therapeutic intervention measure promoting the differentiation of tumor cells rather than killing cells directly by targeting tumor-initiating stem cells. Histone deacetylase (HDAC) inhibitors have been brought into focus as anti-proliferation agents in the differentiation treatment of cancer for their potential to induce cell apoptosis, promote cell differentiation and block the cell cycle. Valproic acid (VPA) represents one of the most efficient broad-spectrum antiepileptic drugs and exerts therapeutic effects on mania as well. Exposure to VPA may increase the risk of autism spectrum disorders (ASD) and teratogenicity. Vahid Haghpanah discovers that VPA, as a histone deacetylase inhibitor, reduces the expression of MMP-2 and MMP-9, declines stem cell markers and tumor aggressiveness, and stimulates the anaplastic thyroid cancer cells to redifferentiate. Relying on this specific function, VPA can be used in the differentiation therapy of highly aggressive and poorly differentiated thyroid cancer [137].

Sevoflurane is an effective volatile anesthetic agent widely used both in adults and in children, with potential neurotoxicity leading to postoperative cognitive dysfunction (POCD) under some circumstances. Y. LI finds that by suppressing the expression of miR-155, sevoflurane inhibits the protein expression of MMP-2 and MMP-9, limits cell migration and invasion, and promotes cell apoptosis in papillary thyroid cancer cells. In a word, sevoflurane can be a good choice for the anesthesia of thyroid cancer surgery to improve the prognosis of patients [135].

Celecoxib is a cyclooxygenase-2 (COX-2) selective suppressor that exhibits anti-tumor potential in various types of tumors, such as intestinal cancer, skin cancer, and breast cancer, and anti-inflammatory potential in various types of arthritis such as osteoarthritis, rheumatoid arthritis, and musculoskeletal arthritis. When applied to the treatment of thyroid cancer, celecoxib decreases the protein and mRNA levels of COX-2, MMP-9, and VEGF in human medullary thyroid cancer TT cells in vitro and in the nude mice model of human medullary thyroid cancer in vivo. As a result, celecoxib inhibits growth and angiogenesis by decreasing the production of prostanoid-like COX-2.

In addition to chemical molecules, there are some other approaches inhibiting the expression of MMP-9 and assisting in the treatment of thyroid carcinoma. Due to advanced ideas and technologies, non-thermal atmosphere pressure plasma (NTP) has been generated at room temperature and applied to multiple fields, such as the prevention of plant diseases, inactivation of bacteria, activation, and proliferation of human adult stem cells, and plasma treatment of a few kinds of tumors. NTP decreases the activity of MMP-2, MMP-9, and uPA, inhibits Akt and ERK signaling, and rearranges the cytoskeleton regulated by the FAK/Src complex resulting in changes in cellular morphology. Under these mechanisms, NTP exerts anti-tumor effects by inhibiting the invasion and metastasis of papillary thyroid cancer cells and opens up a novel strategy to treat invasive and metastatic thyroid carcinoma as a consequence [143].

Seeing the MMP-9 inhibitors applied to thyroid cancer, we find that these kinds of inhibitory molecules get involved in a series of critical steps in the tumor progression, for instance, in proliferation, migration, invasion, metastasis, angiogenesis, apoptosis, redifferentiation and epithelial-mesenchymal transition (EMT).

EMT is a reversible cellular program involved in embryonic development, wound healing, and cancer progression. Under normal situations, epithelial cells are conjoined together laterally to form polar sheets displaying polarity, whereas mesenchymal cells exhibit no conjunctions with adjacent cells, completely on the contrary. In the context of tumors, epithelial tumor cells turn into mesenchymal cells through diminishing cellular conjunctions, decreasing focal adhesion, down-regulating epithelial markers such as E-cadherin and Keratin, up-regulating mesenchymal markers such as N-cadherin and vimentin, increasing cell mobility, remodeling cytoskeleton, and degrading extracellular matrix under the assistance of MMP-9 and other MMPs [144].

Under the background of thyroid cancer, high levels of MMP-9 promote the EMT process, bound up with the invasion, migration, metastasis, and apoptosis of thyroid cancer cells. Inhibition of MMP-9 suppresses the occurrence of EMT and consequently retards the tumor progression, increases the survival time, and improves the survival quality of patients, which further proves the therapeutic abilities of MMP-9 inhibitors used in thyroid cancer.

Non-coding RNAs refer to RNAs that are transcribed from genes but are not translated into proteins, performing their biological functions at the RNA level. MicroRNAs (miRNAs) are a class of non-coding single-stranded RNA molecules which are about 22 nucleotides in length. MiRNAs target tumor suppressors or promoters to adjust the progression of the tumor, exhibiting inhibitory or accelerating effects conversely due to different molecules. MiR-205 declines MMP-9 expression and suppresses angiogenesis and EMT, which can be an alternative therapeutic strategy to treat ATC [145]. In addition, miR-214 [146], miR-203 [147], and several miRNAs have similar abilities. On the contrary, miR-106a-5p increases MMP-9 levels, promotes the activity, migration, and invasion of PTC cells, and inhibits cell apoptosis [148]. Long non-coding RNAs (lncRNAs) have a length of about 200 nucleotides, participating in cell progression and disease development. LncRNA UCA1 has a high level in PTC consistent with high expression of MMP-9, which is related to tumor stages and lymph node metastasis [149]. LncRNA LINC0031 reduces the expression of MMP-9, suppresses the activation of the P13K/Akt signaling pathway, and inhibits tumor progression [150]. Circular RNAs (circRNAs) are produced by the circulation mechanism of exons. CircRNA DOCK1 not only becomes enriched in tumor tissues but also induces the accumulation of MMP-9, contributing to thyroid carcinoma [151].

## 5. Conclusions and Prospects

The review focuses on the role of MMP-9 as a biomarker and MMP-9 inhibition as a treatment method in various diseases, taking thyroid carcinoma as an instance. There are generally high MMP-9 mRNA or protein levels in thyroid tumor tissues, malignant tumor tissues, metastatic tumor tissues, and other samples with a high degree of malignancy rather than a low degree. What is more, high expression of MMP-9 has an intimate relationship with the presence of lymph node metastasis, the presence of distant metastasis, extrathyroid invasion, high degree of tumor infiltration, advanced TNM stage, a large tumor size, the aged population and other clinical factors that exhibit poor prognosis.

The review summarizes and enumerates MMP-9 inhibitors or MMP-9 inhibitory molecules applied to thyroid cancer and other disorders. The molecules can be roughly divided into natural groups and non-natural groups, both of which participate in multiple aspects of diseases and show effective therapeutic effects. Especially in thyroid tumors, natural MMP-9 inhibitors represent comprehensive curative potential in different kinds of thyroid cancer cells from multiple angles, such as inhibition of viability, adhesion, motility, proliferation, migration, invasion, metastasis, angiogenesis and EMT, and promotion of apoptosis and redifferentiation.

The above conclusion principally confirms two points: 1. The expression of MMP-9 increases in various pathological processes, including thyroid cancer. 2. The inhibition of MMP-9 exhibits favorable abilities for treatment in multiple disorders, including thyroid cancer as well. These inhibitory molecules open up a new idea of therapy, and because of their high efficiency, high comprehensiveness, low toxicity, and low side effects, they possess immeasurable clinical values and application prospects after a series of clinical trials and technique improvements. We believe that with the sight into the inhibition of MMP-9, more curative natural and non-natural drugs with high inhibitory properties will be discovered, screened, and designed in the future.

## Figures and Tables

**Table 2:** Non-natural MMP-9 inhibitors and inhibitory molecules applied to different diseases.

Reference	Molecules	Research Methods	Research Diseases	Research Subjects	Effective Dose
[16]	Theissenolactone C (LC53)	Gelatin zymography RT-PCR Western blotting	Glaucoma	MCP-1-stimulated THP-1 cells	2 µM, 5 µM, 10 µM
[16]	Theissenolactone C (LC53)	Gelatin zymography RT-PCR Western blotting	Glaucoma	IL-1β-activated primary astrocytes derived from the rat brain	2 µM, 5 µM, 10 µM
[16]	Theissenolactone C (LC53)	Gelatin zymography RT-PCR Western blotting	Glaucoma	An SD rat model of high intraocular pressure (IOP)-related retinal ischemia reperfusion injury	10 mg/kg
[16]	Memantine	Gelatin zymography RT-PCR Western blotting	Glaucoma	An SD rat model of high intraocular pressure (IOP)-related retinal ischemia reperfusion injury	20 mg/kg
[16]	Memantine	Gelatin zymography RT-PCR Western blotting	Glaucoma	MCP-1-stimulated THP-1 cells	100 µM
[38]	SB-3CT	Gelatin zymography	Embolic focal cerebral ischemia	A C57Bl/6 J mouse model of embolic focal cerebral ischemia	25 mg/kg
[39]	SB-3CT	Immunohistochemistry	Traumatic brain injury (TBI)	An SD rat model of traumatic brain injury induced by a fluid percussion	50 mg/kg
[40]	SB-3CT	RT-PCR	Corneal lymphangiogenesis	A C57BL/6 mouse model of inflammatory corneal neovascularization induced by corneal suture placement	50 µM, 100 µM, 200 µM
[41]	AZD1236	Gelatin zymography	Spinal cord injury	A mouse and rat model of spinal cord injury	100 mg/kg, 200 mg/kg, 300 mg/kg (oral administration) 2.5 mg/mL, 5 mg/mL, 10 mg/mL (intrathecal injection)
[42]	AZD1236	/	Chronic obstructive pulmonary disease (COPD)	Patients with stable moderate-to-severe COPD	75 mg
[43]	AZ11557272	Western blotting	Smoke-induced emphysema	A Hartley strain guinea pig model exposed to the smoke or air	100 mg/kg
[44]	(Ⅰ-3,Ⅱ-3)-biacacetin	Gelatin zymography	Fibrosarcoma	HT1080 cells	10 μM
[45]	AZD3342	ELISA Gelatin zymography RT-PCR	Colonic anastomoses	An SD rat model constructed of colonic anastomoses	50 mg/kg
[46]	GM6001	ImmunohistochemistryWestern blotting	Hypertensive cerebropathy	Dahl salt-sensitive (Dahl/SS) and Lewis rat models fed with a high-salt diet	1.2 mg/kg
[47]	GM6001	Western blotting	Single severe traumatic brain injury (ssTBI)	A C57BL/6 WT mouse model of traumatic brain injury stimulated by a closed head injury	50 mg/kg
[48]	AG-L-66085	RT-PCR	Retinoblastoma (Rb)	Y79 cells Weri-1 cells	5 μM
[49]	(R)-ND-336	Gelatin zymography	Diabetic foot ulcers (DFUs)	A db/db mouse model with a single 8-mm diameter full-thickness excisional wound	2 mg/kg
[50]	(R)-ND-336	Gelatin zymography	Diabetic foot ulcers (DFUs)	A db/db mouse model with an infected wound	10 μg
[51]	β-aminopropionitrile (BAPN)	ELISA Gelatin zymography Western blotting	Gastric carcinoma	A nude mouse model of gastric carcinoma inoculated SGC-7901 cells	0.1 mM, 0.2 mM, 0.3 mM
[52]	Indinavir (IDV)	Gelatin zymography RT-PCR	Cervical carcinoma (CC)	An HPV16/E2 mouse transgenic model of HR-HPV-induced estrogen-promoted CC	1.4 mg/day
[52]	Saquinavir (SQV)	Gelatin zymography RT-PCR	Cervical carcinoma (CC)	An HPV16/E2 mouse transgenic model of HR-HPV-induced estrogen-promoted CC	1 mg/day
[52]	Lopinavir (LPV)	Gelatin zymography RT-PCR	Cervical carcinoma (CC)	An HPV16/E2 mouse transgenic model of HR-HPV-induced estrogen-promoted CC	0.46 mg/day
[53]	Delta-tocotrienol (δT)	Gelatin zymography RT-PCR Western blotting	Non-small-cell lung carcinoma (NSCLC)	A549 cells H1299 cells	10 μM, 20 μM, 30 μM
[54]	Simvastatin	Gelatin zymography	Glaucoma	Primary astrocytes derived from the human optic nerve head	5 μg/mL
[54]	Lovastatin	Gelatin zymography	Glaucoma	Primary astrocytes derived from the human optic nerve head	5 μg/mL
[54]	Atorvastatin	Gelatin zymography	Glaucoma	Primary astrocytes derived from the human optic nerve head	5 μg/mL
[55]	Lipoxin A4 methyl ester (LXA4 ME)	Western blotting	Intracerebral hemorrhage (ICH)	An SD rat model of intracerebral hemorrhage	10 ng/day, 100 ng/day
[56]	Hydroxytyrosol (HT)	ELISA Gelatin zymography RT-PCR	Atherosclerosis (AS)	PBMCs U937 cells	1–10 μM IC50 = 10 μM
[57]	Walnut-derived peptide TWLPLPR (TW-7)	Immunohistochemistry	Alzheimer’s disease (AD)	β-amyloid 25–35-injured bEnd.3 cells	100 μM
[58]	Thiamine	ELISA	Sepsis	86 blood samples of septic patients	200 mg
[58]	Ascorbic acid	ELISA	Sepsis	86 blood samples of septic patients	50 mg/kg
[59]	MK2206 2HCl	ImmunohistochemistryRT-PCR Western blotting	Oral squamous cell carcinoma (OSCC)	CAL27 cells SCC25 cells	6 μM, 10 μM
[60]	U0126	Gelatin zymography RT-PCR	Diabetic retinopathy (DR)	An SD rate model of diabetes induced by streptozotocin	0.1 mM
[61]	BB-94 (Batimastat)	Gelatin zymography RT-PCR	Acute kidney allograft rejection	A Lewis rat model of orthotopic kidney allotransplantation	30 mg/kg
[62]	Doxycycline	Gelatin zymography	Skeletal muscle ischemia-reperfusion injury	An SD rat model of skeletal muscle ischemia-reperfusion injury	50 mg/kg, 200 mg/kg
[63]	Melatonin	Gelatin zymography Western blotting	Transient focal cerebral ischemia	A C57BL/B6 mouse model of transient focal cerebral ischemia	5 mg/kg
[64]	1, 25(OH)2D3	ELISA	Pulmonary tuberculosis (PTB)	Peripheral blood mononuclear cells (PBMCs) from 43 PTB patients	0.1 μM
[65]	Imidaprilat	Gelatin zymography	Atherosclerosis	THP-1 cells	100 nM, 1000 nM

**Table 5:** The signaling pathways in thyroid carcinoma, including MMP-9.

Reference	Type of Thyroid Carcinoma	Signaling Pathways
[107]	PTC	TR4/circ-FNLA/miR-149-5p/MMP-9
[108]	PTC	SOX12/POU2F1, POU3F1/MMP-9
[109]	PTC	DUXAP10/Akt/mTOR/MMP-9
[110]	PTC	KDM1A/TIMP-1/MMP-9
[111]	PTC	ALOX5/ MMP9
[112]	PTC	ROCK1/MMP-9
[113]	FTC	FRNK/FAK/EGF/MMP-9
[114]	FTC	S1P/S1P1/MMP-9
[115]	ATC	PLK1/MMP-9
[116]	ATC	S1P/S1P2/MMP-9
[117]	PTC, FTC	S100A4/MMP-9
[118]	PTC, ATC	Enigma/PI3K/AKT/MMP-9
[119]	PTC, FTC, ATC	IL-17RB/ERK1/2/MMP-9

**Table 6:** MMP-9 inhibitors and inhibitory molecules applied to thyroid carcinoma.

Reference	Molecules	Research Methods	Type of Thyroid Carcinoma	Research Models	Doses	Results
[122]	Curcumin	Gelatin zymography Western blotting	PTC	K1 cells	12.5 μM, 25 μM, 50 μM	Migration inhibition Metastasis inhibition EMT inhibition Viability inhibition
[123]	Curcumin	Gelatin zymography	PTC	K1 cells	25 μM, 50 μM	Migration inhibition
[124]	Curcumin	Western blotting	PTC	TPC-1 cells	23.31 μM	Migration inhibition Invasion inhibition EMT inhibition Viability inhibition
[125]	Evodiamine	Western blotting	PTC	TPC-1 cells	5 μM	Proliferation inhibition Migration inhibition EMT inhibition Viability inhibition
[125]	Evodiamine	Western blotting	ATC	SW1736 cells	5 μM	Proliferation inhibitionMigration inhibitionEMT inhibitionViability inhibition
[126]	Schizandrin A (SchA)	RT-PCR Western blotting	PTC	TPC-1 cells	50 µM	Proliferation inhibition Migration inhibition Invasion inhibition
[127]	Emodin	Western blotting	ATC	8505C cells SW1736 cells	10 µM, 15 µM, 20 µM, 25 µM	Proliferation inhibition Metastasis inhibition Angiogenesis inhibition
[128]	Ginsenoside Rg3	Western blotting	PTC	TPC-1 cells BCPAP cells	50 µM, 100 µM	Metastasis inhibition
[128]	Ginsenoside Rg3	Western blotting	ATC	C643 cells Ocut-2c cells	50 µM, 100 µM	Metastasis inhibition
[129]	Taraxasterol (TAR)	Western blotting	PTC	TPC-1 cells BCPAP cells	2.5 µg/mL, 5 µg/mL, 10 µg/mL	Migration inhibition Invasion inhibition EMT inhibition
[130]	Epigallocatechin-3-gallate (EGCG)	Gelatin zymography Western blotting	PTC	FB-2 cells	10 μM, 40 μM, 60 μM	Proliferation inhibition EMT inhibition Motility inhibition
[131]	Diindolylmethane (DIM)	Gelatin zymography Western blotting	PTC	BCPAP cells	25 µM	Proliferation inhibition Migration inhibition Invasion inhibition Metastasis inhibition Adhesion inhibition
[131]	Diindolylmethane (DIM)	Gelatin zymography Western blotting	ATC	8505C cells	25 µM	Proliferation inhibition Migration inhibition Invasion inhibition Metastasis inhibition Adhesion inhibition
[131]	Diindolylmethane (DIM)	Gelatin zymography Western blotting	FTC	CGTHW-1 cells ML-1 cells	25 µM	Proliferation inhibition Migration inhibition Invasion inhibition Metastasis inhibition Adhesion inhibition
[132]	Salidroside	RT-PCR Western blotting	ATC	WRO cells	10 µM, 20 µM, 40 µM	Migration inhibition Invasion inhibition
[133]	Quercetin	RT-PCR	PTC	BCPAP cells	100 μM	Migration inhibition Invasion inhibition Adhesion inhibition Apoptosis promotion
[134]	Silibinin	Gelatin zymography RT-PCR	PTC	TPC-1 cells	50 µM	Migration inhibition
[135]	Sevoflurane	Western blotting	PTC	TPC-1 cells IHH-4 cells	2.5%	Migration inhibition Invasion inhibition Viability inhibition Apoptosis promotion
[136]	Fingolimod (FTY720)	Gelatin zymography Western blotting	FTC	ML-1 cells	10 μM	Proliferation inhibition Invasion inhibition
[137]	Valproic Acid (VPA)	RT-PCR	ATC	8305C cells	0.1 mM, 1 mM, 5 mM	Redifferentiation promotion
[137]	Valproic Acid (VPA)	RT-PCR	PTC	BCPAP cells	0.1 mM, 1 mM	Redifferentiation promotion
[138]	BMAP-28	Colorimetry RT-PCR	MTC	TT cells	4 µM	Proliferation inhibition
[139]	1α,25(OH)2D3	Gelatin zymography Western blotting	ATC	8505C cells	0.1 µM	Migration inhibition Invasion inhibition
[139]	MART-10	Gelatin zymography Western blotting	ATC	8505C cells	0.1 µM	Migration inhibition Invasion inhibition
[140]	Gemigliptin and metformin	Western blotting	PTC	TPC-1 cells	1 mM Gemigliptin and 30 mM metformin	Proliferation inhibition Migration inhibition Viability inhibition

EMT: epithelial-mesenchymal transition.

## Data Availability

Not applicable.
